# Micro-Geographical Heterogeneity in *Schistosoma mansoni* and *S. haematobium* Infection and Morbidity in a Co-Endemic Community in Northern Senegal

**DOI:** 10.1371/journal.pntd.0002608

**Published:** 2013-12-26

**Authors:** Lynn Meurs, Moustapha Mbow, Nele Boon, Frederik van den Broeck, Kim Vereecken, Tandakha Ndiaye Dièye, Emmanuel Abatih, Tine Huyse, Souleymane Mboup, Katja Polman

**Affiliations:** 1 Institute of Tropical Medicine, Antwerp, Belgium; 2 Laboratory of Bacteriology and Virology, Centre Hospitalier Universitaire Aristide Le Dantec, Dakar, Senegal; 3 Laboratory of Biodiversity and Evolutionary Genomics, University of Leuven, Leuven, Belgium; National Institute of Parasitic Diseases, China

## Abstract

**Background:**

*Schistosoma mansoni* and *S. haematobium* are co-endemic in many areas in Africa. Yet, little is known about the micro-geographical distribution of these two infections or associated disease within such foci. Such knowledge could give important insights into the drivers of infection and disease and as such better tailor schistosomiasis control and elimination efforts.

**Methodology:**

In a co-endemic farming community in northern Senegal (346 children (0–19 y) and 253 adults (20–85 y); n = 599 in total), we studied the spatial distribution of *S. mansoni* and *S. haematobium* single and mixed infections (by microscopy), *S. mansoni*-specific hepatic fibrosis, *S. haematobium*-specific urinary tract morbidity (by ultrasound) and water contact behavior (by questionnaire). The Kulldorff's scan statistic was used to detect spatial clusters of infection and morbidity, adjusted for the spatial distribution of gender and age.

**Principal Findings:**

*Schistosoma mansoni* and *S. haematobium* infection densities clustered in different sections of the community (*p* = 0.002 and *p* = 0.023, respectively), possibly related to heterogeneities in the use of different water contact sites. While the distribution of urinary tract morbidity was homogeneous, a strong geospatial cluster was found for severe hepatic fibrosis (*p* = 0.001). Particularly those people living adjacent to the most frequently used water contact site were more at risk for more advanced morbidity (RR = 6.3; *p* = 0.043).

**Conclusions/Significance:**

*Schistosoma* infection and associated disease showed important micro-geographical heterogeneities with divergent patterns for *S. mansoni* and *S. haematobium* in this Senegalese community. Further in depth investigations are needed to confirm and explain our observations. The present study indicates that local geospatial patterns should be taken into account in both research and control of schistosomiasis. The observed extreme focality of schistosomiasis even at community level, suggests that current strategies may not suffice to move from morbidity control to elimination of schistosomiasis, and calls for less uniform measures at a finer scale.

## Author Summary

In the developing world, over 230 million people are infected with parasitic *Schistosoma* worms. *Schistosoma mansoni* and *S. haematobium* are the most abundant species in Africa, affecting the liver and urinary tract, respectively. Both parasites are spread through infested freshwater. Although it is known that the disease occurs focally within countries or regions, little is known on its geographic spread on a smaller scale. Here, we examined 599 people from a community in northern Senegal for *S. mansoni* and *S. haematobium* infections and related abnormalities of the liver and urinary tract. We recorded where they lived and where they had water contact and visualized this information in geographical maps. The study showed that each *Schistosoma* species clustered in a different section of the community, and that liver abnormalities were more severe near the mostly used water contact site. So far, this is the first study to investigate the geographical spread of both species in a single community, and the first to map schistosomal disease on such a small scale. Further studies are needed to confirm and explain these results. They could contribute to a better understanding of schistosomiasis and have important consequences for the control and elimination of this disease.

## Introduction

Schistosomiasis is amongst the most common human parasitic diseases with over 230 million people affected worldwide [1]. More than 90% of them live in sub-Saharan Africa [2]. The two major species are *Schistosoma mansoni* and *S. haematobium*, which are co-endemic in many regions [3]. However, little is known about the geographical distribution of both species within such co-endemic regions. Knowledge on micro-geographical variations of single and mixed *Schistosoma* infections and associated disease could provide important insights into the drivers of infection and disease and as such better tailor schistosomiasis control and elimination efforts.

Recent progress in geographic information systems (GIS) has facilitated a better understanding of geospatial dimensions of schistosomiasis on the large scale. On continental and national scales, climatic (e.g. temperature and rainfall) and physical factors (e.g. vegetation, large water bodies, altitude) have been identified as major determinants of the heterogeneous geographical distribution of *Schistosoma* infection (either *S. mansoni* or *S. haematobium*, e.g. [4]–[13]). On subnational levels, distance to water contact sites, land use and the distribution of infected snails have been reported to contribute to these heterogeneities (e.g. [14]–[20]).

Few studies have however exploited these techniques to address the geospatial dimensions of schistosomiasis on the micro-scale, i.e. within communities or among households [21]–[29]. Most of these considered spatial patterns of only one *Schistosoma* species even though *S. mansoni* and *S. haematobium* often occur together [3]. Moreover, micro-geographical clustering of *Schistosoma* infection has never been studied in relation to *Schistosoma*-specific morbidity.

In the present study, we set out to investigate the spatial patterns of *S. mansoni* and *S. haematobium* infection and morbidity in a co-endemic community on the bank of Lake Guiers in the north of Senegal [30], [31]. During the past decades, many communities around Lake Guiers (‘Lac de Guiers’) in the north of Senegal have become co-endemic for *S. mansoni* and *S. haematobium* [32]–[35]. *Schistosoma mansoni* was introduced in Richard-Toll in 1988 upon construction of the Diama dam and rapidly spread throughout the region [36], [37]. By 1994, virtually the whole Lake Guiers area had become exposed to this species [38]. Today, both *S. mansoni* and *S. haematobium* are wide-spread in the communities around the lake, and the situation is still dynamic.

## Methods

### Ethics statement

This study was part of a larger investigation on the epidemiology of schistosomiasis and innate immune responses (SCHISTOINIR: www.york.ac.uk/res/schistoinir) for which approval was obtained from the review board of the Institute of Tropical Medicine, the ethical committee of the Antwerp University Hospital and ‘Le Comité National d'Ethique de la Recherche en Santé’ in Dakar. Informed and written consent was obtained from all participants prior to inclusion into the study. For minors, informed and written consent was obtained from the legal guardian.

Participants with severe pathology that needed further treatment were referred to the appropriate health authority. After the study, all community members were offered praziquantel (40 mg/kg) and mebendazole (500 mg) to treat and prevent schistosomiasis and soil-transmitted helminthiasis, respectively [30], according to WHO guidelines [39].

### Study area

**Figure 1:**
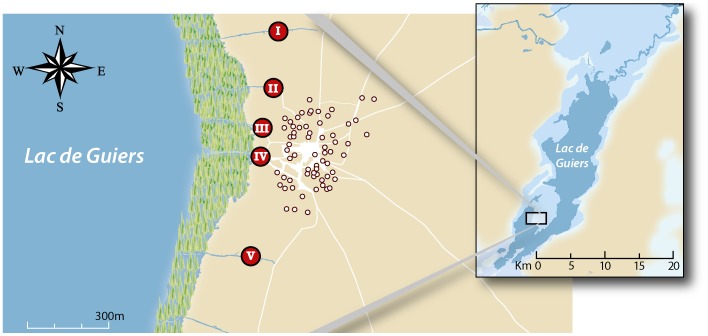
General map of the study village. The inset shows that Diokhor Tack is located in the south-west of the Nouk Pomo peninsula in Lac de Guiers. The main figure indicates the locations of the 68 households that participated in the study (white dots). Red dots with Roman numerals refer to the sites where people come into direct contact with water from the lake. Water contact site III and IV are open spaces enclosed by dense vegetation. Site IV is the largest water contact site. The more remote water contact sites (I, II, and V) are located along irrigation canals in which vegetation was more sparse at the time of study.

This cross-sectional study was conducted from July until November 2009 in Diokhor Tack (16°11′24″N 15°52′48″W), the largest community on the Nouk Pomo peninsula in Lake Guiers. Details on the study area have been described elsewhere [30]. In short, it is an isolated, compact and homogeneous Wolof community of Muslim faith with a surface of ∼0.25 km^2^. Cultivation is the main means of subsistence and the farmlands that surround the village are irrigated with water from the lake. Although the water from Lake Guiers is piped to the capital city of Dakar, 250 km away [40], the people living nearby do not have access to safe water. Water contact takes place in the lake or in specific sites in canals that are connected to the lake in the west (Figure 1). There were no periodic anthelminthic treatment programs prior to our study and the community does not have a health facility. The nearest ‘health post’ is ∼12 km away. All community members that gave informed consent (or their legal guardians) were included in the study. Participants were registered and recruited from door to door for the parasitological and ultrasound surveys. The community consisted of 71 households, 68 of which participated in this study (Figure 1). This corresponded to a total study population of 599 individuals.

### Data collection and definitions

For microscopic diagnosis of *Schistosoma* infection, two feces and two urine samples were collected from each participant on consecutive days. For each feces sample, two Kato-Katz slides of 25 mg fecal material each were prepared, and urine samples were filtered and processed according to standard procedures, as previously described [30], [31]. In analogy with earlier micro-geographic studies [21]–[23], *S. mansoni* and *S. haematobium* infection densities were expressed as the number of eggs detected per gram of feces (epg) or per 10 ml of urine (ep10ml), respectively, including both negative (0 epg or 0 ep10ml) and positive individuals [41]. Single infection was defined as passing eggs of only one species, and mixed infection as passing eggs of both *S. mansoni* and *S. haematobium*, irrespective of the route of egg elimination [30]. *Schistosoma*-specific morbidity was determined by ultrasound, as previously described [31]. Pathologic lesions associated with *S. haematobium* or *S. mansoni* infection were recorded according to the Niamey guidelines [42]. Individuals with signs of hepatic morbidity that were not specific to *S. mansoni* (e.g. hepatitis, cirrhosis or fatty liver) were excluded [42]. To assess the presence or absence of *S. mansoni*-specific hepatic fibrosis, the liver image pattern was determined [42]. Liver image patterns of C (“periportal fibrosis possible”) to F (“very advanced periportal fibrosis”) were categorized as *S. mansoni*-specific hepatic fibrosis [31]. Individuals with liver image pattern A (“no sign of periportal fibrosis”) or B (“incipient periportal fibrosis not excluded”) were categorized as controls [42]. To assess the presence or absence of *S. haematobium*-specific urinary tract morbidity, the urinary bladder score was determined [42]. A score of ≥1 was considered as *S. haematobium*-specific bladder morbidity in accordance with previous studies [31], [43], [44]. The severity of morbidity was represented by the liver image pattern score for *S. mansoni*- and by the upper urinary tract score for *S. haematobium*-specific morbidity [42]. Finally, individual questionnaires were used to explore water contact behavior in a random subsample of people older than 5 years of age.

### Mapping and geospatial processing

Water contact sites as well as the center of each household were located using a hand-held differential global positioning system with an accuracy of 3 m (Garmin Etrex H). Household locations in latitude and longitude were then linked to the collected individual infection, morbidity and questionnaire data (multiple observations per location). These data were imported into SaTScan 9.1.1 (Software for the spatial and space-time scan statistics, developed by M. Kulldorff, Harvard Medical School, Boston and Information Management Services Inc., Silver Spring, Maryland, USA. Available at www.satscan.org) according to the software's user guide [45]. ArcMap 9.3 (ESRI, Redlands, California, USA) was used to project the geographic coordinates and statistically significant clusters (see below) on to the Universal Transverse Mercator zone 28N (1984 datum).

### Spatial statistics

The widely used Kulldorff's scan statistic in SaTScan™ tests whether events such as disease cases are distributed randomly in space and, if not, identifies the approximate location of significant geospatial clusters [46]. The test uses a moving circular window that varies up to a predefined size. Each window is a potential cluster. For each window, a likelihood ratio test is applied based on the observed and expected number of cases inside and outside the window to test the null hypothesis of absolute spatial randomness against the alternative hypothesis that there is an elevated risk within the window as compared to outside. The window with the maximum likelihood is the ‘most likely cluster’. The *p*-value of the maximum likelihood ratio test statistic was obtained after 999 Monte Carlo replications. A maximum window size of 50% of the study population was chosen upon sensitivity analysis using maximum sizes from 10 to 50%. Only statistically significant (*p*<0.05) most likely clusters were reported, and standard settings (i.e. non-overlapping secondary clusters) were used throughout all analyses. In case the most likely (significant) cluster contained only one household, an additional check was performed to increase the robustness of cluster detection. The standard analysis was repeated while allowing for overlapping secondary clusters (using the “criteria for reporting secondary clusters” option “no restriction = most likely cluster for each grid point” [45]), and the secondary cluster (including the first household) was reported, if it remained significant. Additionally, clusters with *p*<0.06 were displayed to indicate households that tended to have increased risks.

Infection densities of *S. mansoni* and *S. haematobium* showed skewed distributions, and were therefore normalized by log (base 10)-transformation after adding half of the detection limit to allow for zeros. The detection limit for *S. mansoni* infection was 10 epg and that for *S. haematobium* infection 0.5 ep10ml. The spatial distribution of log-transformed infection densities was assessed using normal models [47]. Geometric mean (GM) infection densities in- and outside spatial clusters were computed to quantify significant spatial heterogeneities. Subsequently, Bernoulli models [46] were run to investigate the distribution of single *S. mansoni*, single *S. haematobium* and mixed infections, comparing spatial distributions of people with:

- single *S. mansoni* (1) *versus* those without single *S. mansoni* infections (0);
- single *S. haematobium* (1) *versus* those without single *S. haematobium* infections (0);
- mixed (1) *versus* those without mixed infections (0).

The spatial distribution of the prevalence of hepatic fibrosis and urinary tract morbidity was tested using binary variables in separate Bernoulli models. Ordinal models were used to assess the distribution of the severity of *S. mansoni*- and *S. haematobium*-specific morbidity [48]. Relative risks (RR) comparing people in- and outside clusters, as well as prevalences in- and outside clusters were calculated to quantify significant spatial heterogeneities based on Bernoulli and ordinal models.

Gender and age are important risk factors for both *Schistosoma* infection [30], and morbidity [31]. To investigate whether these demographic factors 1) caused clustering of infection and morbidity, and/or 2) impacted on the size and exact locations of statistically significant clusters, the abovementioned analyses were adjusted using multiple datasets [49]. Six datasets were prepared, containing either males or females from 0 to 9, 10 to 19, or ≥20 years old (Table 1). SaTScan™ incorporated all datasets into a single log likelihood function. This function is defined as the sum of the individual log likelihoods for those data sets for which the observed case count is more than the expected. Since this adjustment was only possible for the Bernoulli and normal models, separate Bernoulli models were run for the ordinal morbidity model showing significant spatial heterogeneities in the unadjusted analysis.

**Table 1:** Characteristics of the 6 datasets used for the gender- and age-adjusted spatial analyses.

		Infection^a^				Morbidity^a^		
Gender	Age (years)	n_total_	Single *S. mansoni*	Single *S. haematobium*	Mixed infections	n_total_	Hepatic fibrosis	Urinary tract morbidity
Male	0–9	109	15	17	27	49	3	41
Female	0–9	79	9	16	17	28	2	18
Male	10–19	90	21	10	49	49	12	48
Female	10–19	68	11	6	42	34	6	26
Male	≥20	103	39	3	15	49	33	44
Female	≥20	150	44	20	39	82	34	56
**Total**		**599**	**139**	**72**	**189**	**291**	**90**	**233**

^a^ Total study population in the first columns and numbers of cases in subsequent columns.

Finally, Bernoulli models were used to compare the geospatial distribution of people reporting to frequent a particular water contact site *versus* the distribution of people who did not report to frequent that site.

## Results

### Characteristics of the study population

Complete parasitological data were obtained from a total of 599 individuals from 68 households. The median household size was 8 people (range 1–20). The total study population consisted of 302 males and 297 females with a median age of 15 (range 0–85) years. Ultrasound and questionnaire data were obtained from random subsamples of 291 individuals (64 households), and 277 individuals (63 households), respectively. The prevalence of overall *S. mansoni* infection was 55% (328/599) and that of *S. haematobium* 44% (261/599). Mixed infections were observed in 32% of the population (189/599). The prevalence of *S. mansoni*-specific hepatic fibrosis was 31% (90/291). Most cases had liver image pattern C (71/90), 9/90 had pattern D, while advanced periportal fibrosis was observed in 10/90 cases (nine with liver image pattern E and one with F). The prevalence of *S. haematobium*-specific urinary tract morbidity was 80% (233/291). Positive upper urinary tract scores (range 3–12) were observed in 6% of the study population (18/291). Distributions of single and mixed *Schistosoma* infections, *S. mansoni*-associated hepatic fibrosis and *S. haematobium*-associated bladder morbidity according to gender and age are summarized in Table 1.

### Spatial distribution of *Schistosoma* infection

**Figure 2:**
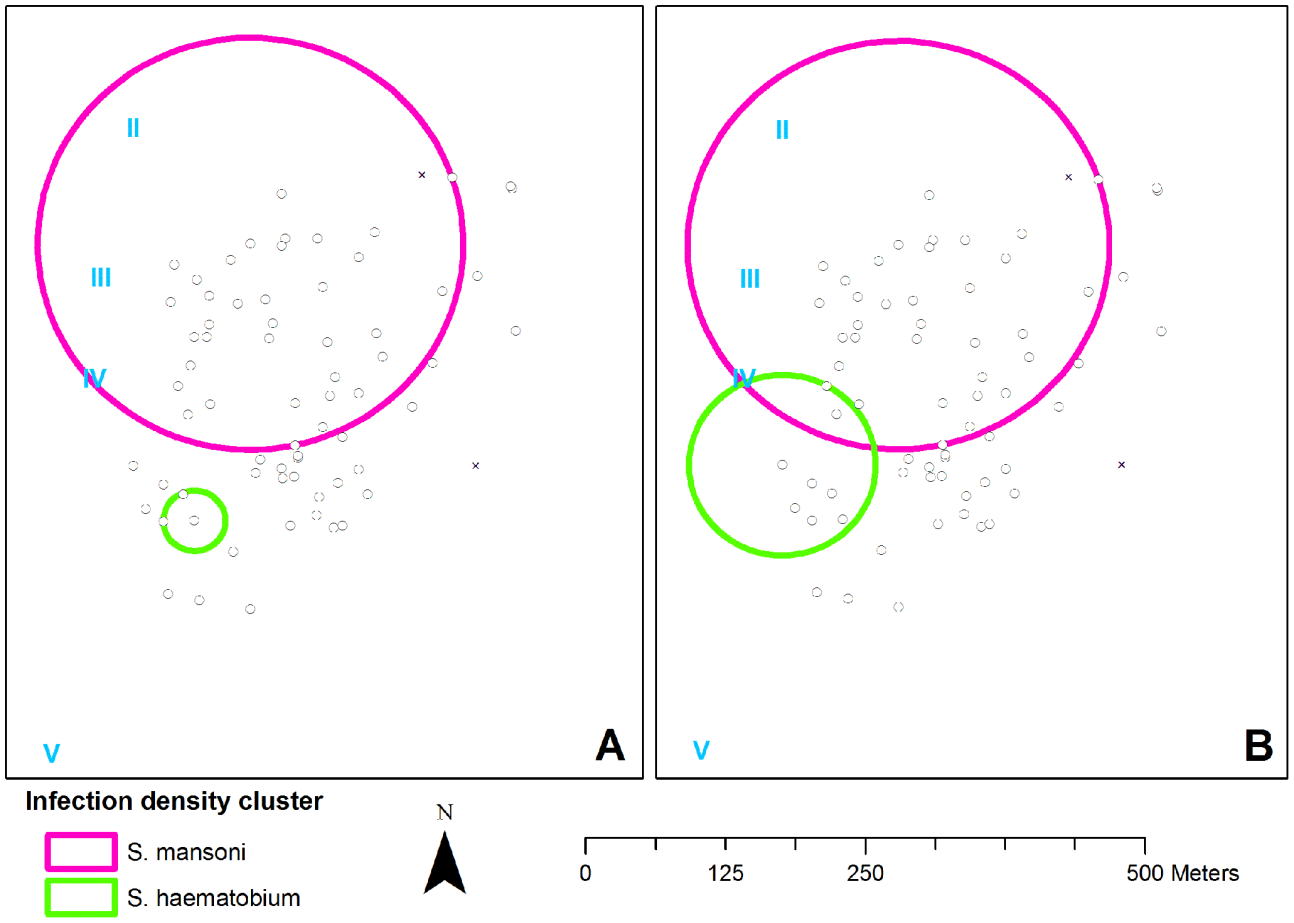
Spatial distribution of *S. mansoni* and *S. haematobium* infection densities. Black circles and crosses indicate households that were included in and excluded from the analysis, respectively. Roman numerals indicate water contact sites. **Panel A** depicts the unadjusted clusters (*p* = 0.001 for both *S. mansoni* and *S. haematobium*). The geometric mean (GM) *S. mansoni* infection density was 33 epg for those living in inside the northern *S. mansoni* cluster (n = 285) compared to12 epg in the rest of the community (n = 314). The GM *S. haematobium* infection density was 4.7 ep10ml inside the southern *S. haematobium* cluster (n = 34) and 0.7 ep10ml outside (n = 565). **Panel B** depicts the gender- and age-adjusted clusters (*p* = 0.002 for *S. mansoni* (north), and *p* = 0.023 for *S. haematobium* (south).

Figure 2A depicts the heterogeneous geospatial distribution of *S. mansoni* and *S. haematobium* infection densities (*p* = 0.001 for both unadjusted analyses). While the size of the *S. haematobium* infection density cluster increased upon correction for the spatial distribution of gender and age, both *S. mansoni* and *S. haematobium* clusters remained statistically significant (Figure 2B; *p* = 0.002 and *p* = 0.023, respectively).

**Figure 3:**
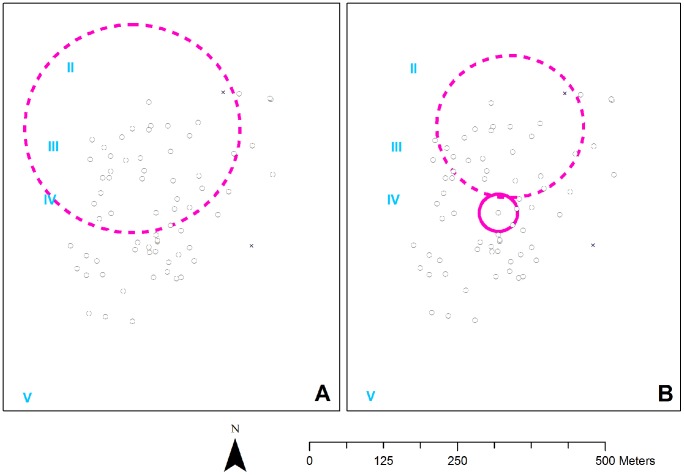
Spatial distribution of single *S. mansoni* infections. Black circles and crosses indicate households that were included in and excluded from the analysis, respectively. Blue Roman numerals indicate water contact sites. Continuous pink circles are statistically significant clusters (*p*<0.05) and dotted circles are borderline significant (*p*<0.06). **Panel A** depicts the unadjusted cluster (RR = 1.7; *p* = 0.053). The prevalence of single *S. mansoni* infection was 30% (83/278) inside and 17% (56/321) outside the cluster. **Panel B** shows the gender- and age-adjusted clusters (*p* = 0.045 for the most likely cluster (center) and *p* = 0.050 for the secondary cluster (north).

Participants with mixed and those with single *S. haematobium* infections were randomly distributed (*p* = 0.16 and *p* = 0.080, respectively), while those with single *S. mansoni* infections tended to cluster geographically (Figure 3A; RR = 1.7; *p* = 0.053). Figure 3B indicates that the clustering of single *S. mansoni* was independent of the spatial distribution of gender and age (*p*≤0.050), although the cluster size and exact location were slightly altered upon adjustment.

### Spatial distribution of *Schistosoma*-specific morbidity

**Figure 4:**
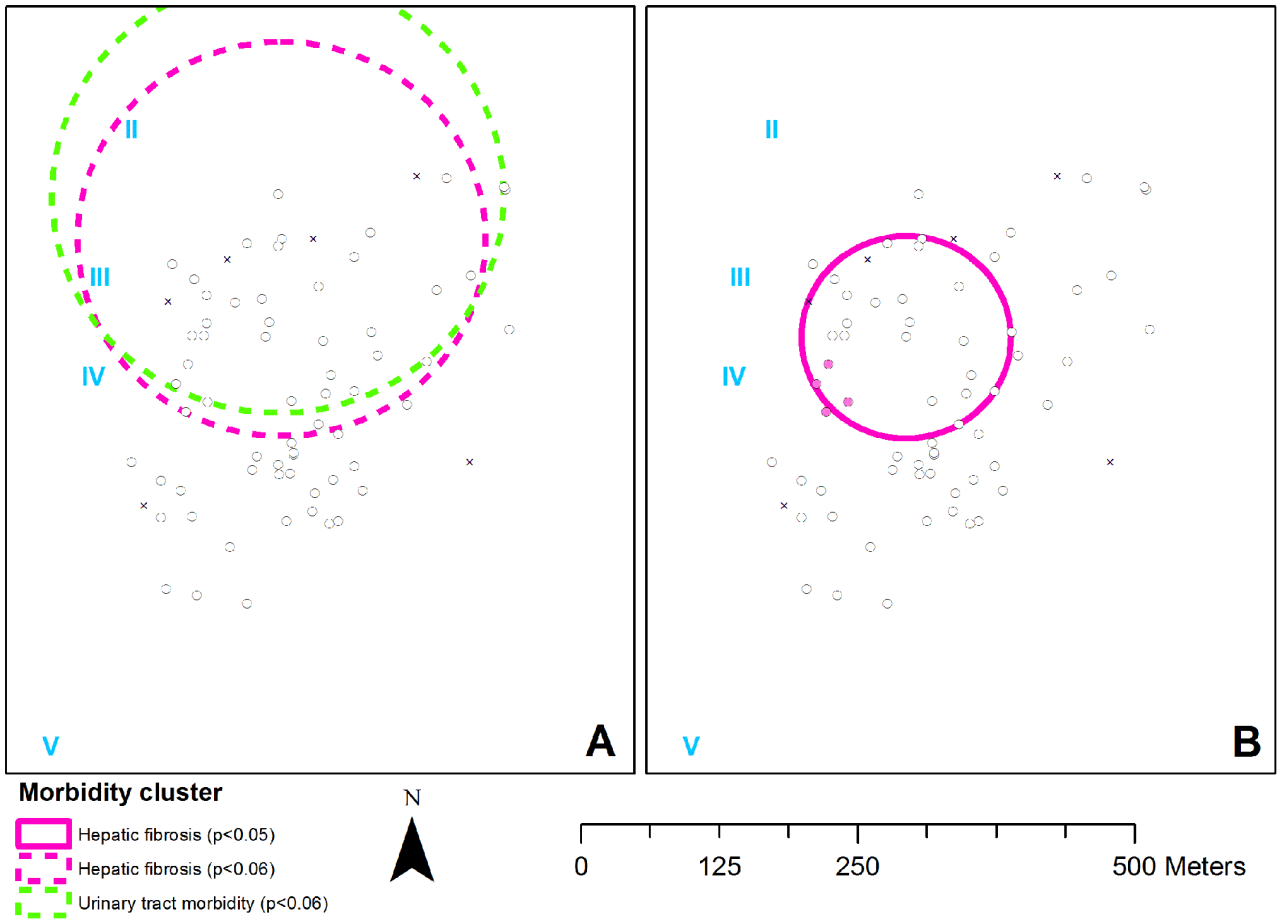
Spatial distribution of *S. mansoni*- and *S. haematobium*-specific morbidity. Black circles and crosses indicate households that were included and excluded from the analysis, respectively. Continuous colored circles are statistically significant clusters (*p*<0.05) and dotted circles are borderline significant (*p*<0.06). Roman numerals indicate water contact sites. **Panel A** depicts the unadjusted clusters for the prevalence of morbidity. Both *S. mansoni*-specific hepatic fibrosis (pink dotted circle; RR = 1.9) and *S. haematobium*-specific urinary tract morbidity clusters (green dotted circle; RR = 1.2) were borderline significant (*p* = 0.054 and *p* = 0.053, respectively). The prevalence of hepatic fibrosis was 41% (59/145) in- and 21% (31/146) outside, and that of urinary tract morbidity was 89% (117/131) in- and 73% (116/160) outside the cluster. Gender- and age-adjusted analysis revealed no (borderline) significant clusters for the prevalence of morbidity. **Panel B** depicts the clusters of morbidity by severity. The risk of severe hepatic fibrosis was elevated in the circle with a RR of 0.3 for liver image pattern A, 1.3 for B, 1.4 for C, 2.7 for D and 4.3 for E, and the only person with pattern F lived here (*p* = 0.001; unadjusted ordinal model). The gender- and age-adjusted cluster for patterns D–F (as opposed to A–C) in adults constituted of the households indicated in pink (*p* = 0.031; Bernoulli model).

Figure 4A indicates that people with hepatic fibrosis (RR = 1.9; *p* = 0.054) and urinary tract morbidity (RR = 1.2; *p* = 0.053) tended to cluster in the same area. Adjusted analysis however indicated that these heterogeneous patterns were dependent on the distribution of gender and age (*p* = 0.087 for hepatic fibrosis and *p* = 0.071 for urinary tract morbidity in the adjusted analysis). In order to assess the distribution of morbidity by severity, ordinal analyses were performed for liver image pattern and upper urinary tract scores. Figure 4B shows that this resulted in one significant cluster (*p* = 0.001) in which the RR increased with the severity of hepatic fibrosis: the RR for a healthy liver image pattern A was 0.3, that for B 1.3, for C 1.4, for D 2.7 and for E 4.3. Moreover, the only person with pattern F in the community lived in this cluster. Bernoulli models were used to investigate whether this cluster of severe hepatic fibrosis was independent of the distribution of gender and age. Since more severe hepatic fibrosis was only observed in adults, these analyses were restricted to ≥20-year-olds. Unadjusted Bernoulli models revealed that image patterns D–F (as opposed to A–C) clustered in the households within the hepatic fibrosis cluster that were closest to water contact site IV (RR = 6.3; *p* = 0.043; data not shown). The combined distribution of patterns E and F (as opposed to A–D) was homogeneous (*p* = 0.20). In the adjusted model, the cluster of pattern D–F remained statistically significant (Figure 4B; *p* = 0.031).

The risk of severe urinary tract morbidity was homogeneously distributed (*p* = 0.38 in the ordinal analysis).

### Water contact behavior

**Figure 5:**
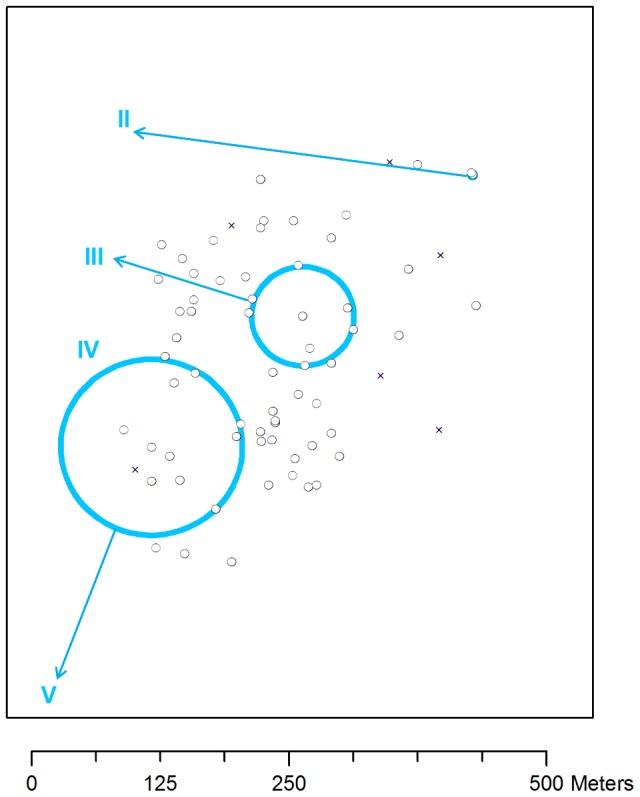
Spatial distribution of self-reported use of the different water contact sites. Black circles and crosses indicate households that were included and excluded from the analysis, respectively. Roman numerals indicate water contact sites. Blue circles indicate clusters of people that reported to frequent a given water contact site indicated by an arrow. Participants in the northeastern cluster were more likely to frequent site II (3/4 *versus* 5/273;*p* = 0.005), participants in the middle cluster to frequent site III (4/17 *versus* 2/260; *p* = 0.022), and those from the southwestern cluster to frequent site V (8/53 *versus* 1/224; *p* = 0.001) than those living outside the respective clusters.

Water contact activities were concentrated at site IV with 62% of the interviewees (172/277) reporting to frequent this site. Numbers of observations in the other sites were limited. Nonetheless, spatial analysis of the questionnaire data revealed significant heterogeneities in the self-reported use of the different water contact sites (Figure 5). People from two adjacent households in the northeast were more likely to frequent site II than those from the rest of the community (3/4 in- *versus* 5/273 outside the cluster; *p* = 0.005). People living in the center of the community were more likely to frequent site III than others (4/17 *versus* 2/260; *p* = 0.022). Those from the southwest were more likely to frequent site V (8/53 *versus* 1/224; *p* = 0.001). Use of site I and IV did not appear to be linked to a particular group of households (*p* = 0.31 and *p* = 0.16 respectively).

## Discussion

The present micro-geographical study revealed significant clusters of *S. mansoni* and *S. haematobium* infection density in different sections of one community in a co-endemic area, possibly related to heterogeneities in the use of different water contact sites. While the distribution of urinary tract morbidity was homogeneous, a strong geospatial cluster was found for severe hepatic fibrosis. Particularly those people living adjacent to the most frequently used water contact site were more at risk for advanced morbidity than those living farther away.

These findings confirm the well-known focality of schistosomiasis [50]. Even within one community, one cannot assume the risk of schistosomiasis to be homogenous. More remarkably even, the two *Schistosoma* species clustered in different sections of the community; *Schistosoma mansoni* infections clustered in the north while *S. haematobium* clustered in the south. A series of recent GIS studies showed significant micro-geographical heterogeneities in *S. haematobium* infection within a mono-endemic Kenyan community [21], [22]. Those in *S. mansoni* mono-endemic communities showed conflicting results with heterogeneous spatial patterns in some studies and homogeneous patterns in others [24]–[29]. To our knowledge, only Farooq *et al* have so far investigated the spatial distribution of both infections in a co-endemic community in Egypt in the 1960s. They reported higher infection levels of *S. mansoni* in small children in one section of the community and higher levels of *S. haematobium* in another section [51]. This is in agreement with the divergent distributions of *S. mansoni* and *S. haematobium* infection densities observed in the present study. Several interrelated factors may underlie these observations, and are discussed below.

First of all, the micro-geographic distributions of the intermediate snail host of *S. mansoni* and *S. haematobium*, which belong to the genus *Biomphalaria* and *Bulinus*, respectively, may be divergent as well. Unfortunately, it was logistically impossible to collect snails in the present study. Yet, it is known that these snail species prefer different niches and that their distribution is influenced by chemical, physical, and biological factors [52]–[57]. Indeed, Woolhouse and Chandiwana demonstrated that the snail hosts of *S. mansoni* and *S. haematobium* occupy different locations in one single habitat in a co-endemic focus [53]. Ecological factors may thus have favored *S. mansoni* transmission in the north and *S. haematobium* transmission in the south.

At the human host level, behavioral factors may have played a role in the observed spatial pattern of infection. Although based on a small number of observations, our questionnaire data indeed indicated that people from the north and center were more likely to frequent the northern sites than other community members, whereas those from the southwest were more likely to use the southernmost site. It thus seems that the first group maintained *S. mansoni* transmission in the north and the second *S. haematobium* transmission in the south. This corresponds to the study of Woolhouse and Chandiwana reporting 1) a similar geospatial segregation of *S. mansoni* and *S. haematobium* infection in the snail host population between transmission sites, and 2) a very focal man-to-snail transmission, within a distance of 40 m. Interestingly, they proposed that these divergent patterns most likely reflected differences in the distribution of defecation from that of urination, favoring *S. mansoni* and *S. haematobium* transmission, respectively [53]. In contrast to water contact behavior, age and gender of the human host were shown to have a negligible impact on the divergent pattern of *S. mansoni* and *S. haematobium* infection. Spatial clustering in the different sections of the community remained significant upon correction for age and gender. Other factors that may have contributed to the spatial pattern include genetic differences in susceptibility to infection [58]–[60]. Indeed, extended families tended to live together in this community (L. Meurs, personal observation). Also, people from the same section/extended family are more likely to have similar behavioral patterns [51], [61]–[63].

In contrast to the spatial distribution of *S. haematobium* infection, the distribution of *S. haematobium*-associated urinary tract morbidity was homogeneous. This was unexpected as *S. haematobium* infection has consistently been reported as an independent risk factor for urinary tract morbidity [43], [44], [64]–[71]. The fact that *S. haematobium* was only introduced in this region approximately 6 years prior to this study [72], may explain the relatively low severity of urinary tract morbidity in this community and the consequent absence of a spatial pattern. The severity of urinary tract morbidity is expected to progress over time with cumulative exposure to *S. haematobium* eggs [73].

On the other hand, a strong geospatial cluster was found for severe *S. mansoni*-specific hepatic fibrosis which overlapped with that of *S. mansoni* infection density. At first sight, this seems to be in contrast with previous studies by this group showing that current *S. mansoni* infection is not associated with hepatic fibrosis [31], which usually develops after 5–15 years of exposure [3]. However, a closer look at the overlapping clusters showed that teenagers had the highest infection densities and contributed most to the *S. mansoni* infection density cluster (data not shown). Adults on the other hand had more advanced morbidity, and contributed most to the severe hepatic fibrosis cluster. This suggests that these adults were in fact the teenagers with the highest *S. mansoni* infection densities earlier in life.

Moreover, the clustering of severe hepatic fibrosis in adults seemed to be associated with the distance to the water. Those living within ∼100 m of the major water contact site (Figure 4B) were at least six times more likely to develop advanced hepatic fibrosis (liver image pattern D–F) than those living further away. However, other factors cannot be excluded such as genetic predisposition [74], diet or nutritional status [75], or co-infections, which may have put those living in close vicinity of the water at a higher risk of developing hepatic morbidity than the rest of the community.

To our knowledge, only Booth *et al* have so far investigated micro-geographical variations in *Schistosoma*-associated morbidity. They found an association between splenomegaly and the combined exposure to *S. mansoni* and *Plasmodium falciparum* but did not explicitly investigate spatial clustering [76].

To our knowledge this is the first study to quantify micro-geographical infection patterns of *S. mansoni* and *S. haematobium* in a co-endemic community, and the first to relate these to patterns of *Schistosoma*-specific morbidity. Apart from the strengths, it is also important to address some limitations of our study. First, the study was cross-sectional and the results were merely descriptive in nature. The present study was a first attempt to describe patterns of schistosome infection and morbidity on a micro-scale, and it was not designed to explain the underlying mechanisms of potential micro-geographical clustering. Based on the limited data that were available, we generated a number of hypotheses, but other risk factors, including environmental, malacological, genetic, immunological and socio-economic factors, should be included in future studies. In addition, more spatial as well as spatio-temporal studies [21]–[23] are necessary to confirm our observations in other geographical areas and to explain them. Another limitation was that there is as yet no standard technique available to investigate spatial patterns. The emergence of various statistical methods has greatly boosted geospatial studies on schistosomiasis and increased our understanding of this disease. On the other hand, the large variety of methods has also hampered the comparison between the different micro-geographical studies that have be conducted so far and standardization is recommended.

Current WHO schistosomiasis control strategies aim to prevent morbidity in later life through regular mass drug administration (MDA) to at risk populations in so-called homogeneous ecological zones [77], [78]. However, the strong micro-geographical clustering of infection and morbidity observed in the present study suggests that less uniform strategies should be developed to better tailor control efforts at the local level. A more targeted approach will be even more relevant in view of resolution WHA65.21 on the elimination of schistosomiasis, recently adopted by the WHO [77]. It is expected that MDA alone cannot break the *Schistosoma* life cycle and that complementary interventions will have to be put in place [79]. Micro-geographical studies will help to get much needed insights into local transmission dynamics of *S. mansoni* and *S. haematobium* and hence to develop sustainable control and elimination strategies [80].

## Supporting Information

Checklist S1 STROBE Checklist.
(DOC)

## References

[1] WHO (2010 February) Schistosomiasis fact sheet. http://www.who.int/mediacentre/factsheets/fs115/en/index.html.

[2] Hotez PJ, Kamath A (2009) Neglected tropical diseases in sub-saharan Africa: review of their prevalence, distribution, and disease burden. PLoS Negl Trop Dis 3: e412. 19707588 10.1371/journal.pntd.0000412 PMC2727001

[3] Gryseels B, Polman K, Clerinx J, Kestens L (2006) Human schistosomiasis. Lancet 368: 1106–1118. 16997665 10.1016/S0140-6736(06)69440-3

[4] Simoonga C, Kazembe LN, Kristensen TK, Olsen A, Appleton CC, et al (2008) The epidemiology and small-scale spatial heterogeneity of urinary schistosomiasis in Lusaka province, Zambia. Geospat Health 3: 57–67. 19021109 10.4081/gh.2008.232

[5] Handzel T, Karanja DM, Addiss DG, Hightower AW, Rosen DH, et al (2003) Geographic distribution of schistosomiasis and soil-transmitted helminths in Western Kenya: implications for anthelminthic mass treatment. Am J Trop Med Hyg 69: 318–323. 14628951

[6] Tsang VC, Hillyer GV, Noh J, Vivas-Gonzalez BE, Ahn LH, et al (1997) Geographic clustering and seroprevalence of schistosomiasis in Puerto Rico (1995). Am J Trop Med Hyg 56: 107–112. 9063371 10.4269/ajtmh.1997.56.107

[7] Brooker S, Clements AC (2009) Spatial heterogeneity of parasite co-infection: Determinants and geostatistical prediction at regional scales. Int J Parasitol 39: 591–597. 19073189 10.1016/j.ijpara.2008.10.014 PMC2644303

[8] Clements AC, Bosque-Oliva E, Sacko M, Landoure A, Dembele R, et al (2009) A comparative study of the spatial distribution of schistosomiasis in Mali in 1984–1989 and 2004–2006. PLoS Negl Trop Dis 3: e431. 19415108 10.1371/journal.pntd.0000431 PMC2671597

[9] Kloos H, Lo CT, Birrie H, Ayele T, Tedla S, et al (1988) Schistosomiasis in Ethiopia. Soc Sci Med 26: 803–827. 3131881 10.1016/0277-9536(88)90174-8

[10] Dennis E, Vorkpor P, Holzer B, Hanson A, Saladin B, et al (1983) Studies on the epidemiology of schistosomiasis in Liberia: the prevalence and intensity of schistosomal infections in Bong County and the bionomics of the snail intermediate hosts. Acta Trop 40: 205–229. 6138973

[11] Ahmed ES, Daffalla A, Christensen NO, Madsen H (1996) Patterns of infection and transmission of human schistosomiasis mansoni and schistosomiasis haematobium in White Nile Province, Sudan. Ann Trop Med Parasitol 90: 173–180. 8762407 10.1080/00034983.1996.11813041

[12] Raso G, Matthys B, N'goran EK, Tanner M, Vounatsou P, et al (2005) Spatial risk prediction and mapping of Schistosoma mansoni infections among schoolchildren living in western Cote d'Ivoire. Parasitology 131: 97–108. 16038401 10.1017/s0031182005007432

[13] Brooker S, Miguel EA, Waswa P, Namunyu R, Moulin S, et al (2001) The potential of rapid screening methods for Schistosoma mansoni in western Kenya. Ann Trop Med Parasitol 95: 343–351. 11454244 10.1080/00034980120063437

[14] Odiere MR, Opisa S, Odhiambo G, Jura WG, Ayisi JM, et al (2011) Geographical distribution of schistosomiasis and soil-transmitted helminths among school children in informal settlements in Kisumu City, Western Kenya. Parasitology 138: 1569–1577. 21679486 10.1017/S003118201100059X

[15] Gazzinelli A (2006) The spatial distribution of Schistosoma mansoni infection before and after chemotherapy in the Jequitinhonha Valley in Brazil. Mem Inst Oswaldo Cruz 101 Suppl 1:63–71: 63–71. 17308749 10.1590/s0074-02762006000900010

[16] Gryseels B, Nkulikyinka L (1988) The distribution of Schistosoma mansoni in the Rusizi plain (Burundi). Ann Trop Med Parasitol 82: 581–590. 3151430 10.1080/00034983.1988.11812294

[17] Chandiwana SK, Taylor P, Clarke VD (1988) Prevalence and intensity of schistosomiasis in two rural areas in Zimbabwe and their relationship to village location and snail infection rates. Ann Trop Med Parasitol 82: 163–173. 3140747 10.1080/00034983.1988.11812224

[18] Gryseels B, Nkulikyinka L (1990) The morbidity of schistosomiasis mansoni in the highland focus of Lake Cohoha, Burundi. Trans R Soc Trop Med Hyg 84: 542–547. 2128666 10.1016/0035-9203(90)90033-b

[19] Kapito-Tembo AP, Mwapasa V, Meshnick SR, Samanyika Y, Banda D, et al (2009) Prevalence distribution and risk factors for Schistosoma hematobium infection among school children in Blantyre, Malawi. PLoS Negl Trop Dis 3: e361. 19156193 10.1371/journal.pntd.0000361 PMC2614474

[20] Coutinho EM, Abath FG, Barbosa CS, Domingues AL, Melo MC, et al (1997) Factors involved in Schistosoma mansoni infection in rural areas of northeast Brazil. Mem Inst Oswaldo Cruz 92: 707–715. 9566243 10.1590/s0074-02761997000500027

[21] Clennon JA, King CH, Muchiri EM, Kariuki HC, Ouma JH, et al (2004) Spatial patterns of urinary schistosomiasis infection in a highly endemic area of coastal Kenya. Am J Trop Med Hyg 70: 443–448. 15100462

[22] Clennon JA, Mungai PL, Muchiri EM, King CH, Kitron U (2006) Spatial and temporal variations in local transmission of Schistosoma haematobium in Msambweni, Kenya. Am J Trop Med Hyg 75: 1034–1041. 17172362

[23] Mutuku FM, King CH, Bustinduy AL, Mungai PL, Muchiri EM, et al (2011) Impact of drought on the spatial pattern of transmission of Schistosoma haematobium in coastal Kenya. Am J Trop Med Hyg 85: 1065–1070. 22144445 10.4269/ajtmh.2011.11-0186 PMC3225153

[24] Stothard JR, Sousa-Figueiredo JC, Betson M, Seto EY, Kabatereine NB (2011) Investigating the spatial micro-epidemiology of diseases within a point-prevalence sample: a field applicable method for rapid mapping of households using low-cost GPS-dataloggers. Trans R Soc Trop Med Hyg 105: 500–506. 21714979 10.1016/j.trstmh.2011.05.007 PMC3183225

[25] Yiannakoulias N, Wilson S, Kariuki HC, Mwatha JK, Ouma JH, et al (2010) Locating irregularly shaped clusters of infection intensity. Geospat Health 4: 191–200. 20503188 10.4081/gh.2010.200

[26] Utzinger J, Muller I, Vounatsou P, Singer BH, N'goran EK, et al (2003) Random spatial distribution of Schistosoma mansoni and hookworm infections among school children within a single village. J Parasitol 89: 686–692. 14533674 10.1645/GE-75R

[27] Pullan RL, Bethony JM, Geiger SM, Cundill B, Correa-Oliveira R, et al (2008) Human helminth co-infection: analysis of spatial patterns and risk factors in a Brazilian community. PLoS Negl Trop Dis 2: e352. 19104658 10.1371/journal.pntd.0000352 PMC2602736

[28] Matthys B, Tschannen AB, Tian-Bi NT, Comoe H, Diabate S, et al (2007) Risk factors for Schistosoma mansoni and hookworm in urban farming communities in western Cote d'Ivoire. Trop Med Int Health 12: 709–723. 17550468 10.1111/j.1365-3156.2007.01841.x

[29] Brooker S, Alexander N, Geiger S, Moyeed RA, Stander J, et al (2006) Contrasting patterns in the small-scale heterogeneity of human helminth infections in urban and rural environments in Brazil. Int J Parasitol 36: 1143–1151. 16814294 10.1016/j.ijpara.2006.05.009 PMC1783908

[30] Meurs L, Mbow M, Vereecken K, Menten J, Mboup S, et al (2012) Epidemiology of mixed Schistosoma mansoni and Schistosoma haematobium infections in northern Senegal. Int J Parasitol 42: 305–311. 22366733 10.1016/j.ijpara.2012.02.002

[31] Meurs L, Mbow M, Vereecken K, Menten J, Mboup S, et al (2012) Bladder Morbidity and Hepatic Fibrosis in Mixed Schistosoma haematobium and S. mansoni Infections: A Population-Wide Study in Northern Senegal. PLoS Negl Trop Dis 6: e1829. 23029589 10.1371/journal.pntd.0001829 PMC3459828

[32] De Clercq D, Vercruysse J, Picquet M, Shaw DJ, Diop M, et al (1999) The epidemiology of a recent focus of mixed Schistosoma haematobium and Schistosoma mansoni infections around the ‘Lac de Guiers’ in the Senegal River Basin, Senegal. Trop Med Int Health 4: 544–550. 10499077 10.1046/j.1365-3156.1999.00444.x

[33] Huyse T, Webster BL, Geldof S, Stothard JR, Diaw OT, et al (2009) Bidirectional introgressive hybridization between a cattle and human schistosome species. PLoS Pathog 5: e1000571. 19730700 10.1371/journal.ppat.1000571 PMC2731855

[34] Southgate V, Tchuem Tchuente LA, Sene M, De Clercq D, Theron A, et al (2001) Studies on the biology of schistosomiasis with emphasis on the Senegal river basin. Mem Inst Oswaldo Cruz 96 Suppl: 75–78. 11586429 10.1590/s0074-02762001000900010

[35] Ten Hove RJ, Verweij JJ, Vereecken K, Polman K, Dieye L, et al (2008) Multiplex real-time PCR for the detection and quantification of Schistosoma mansoni and S. haematobium infection in stool samples collected in northern Senegal. Trans R Soc Trop Med Hyg 102: 179–185. 18177680 10.1016/j.trstmh.2007.10.011

[36] Talla I, Kongs A, Verle P (1992) Preliminary study of the prevalence of human schistosomiasis in Richard-Toll (the Senegal river basin). Trans R Soc Trop Med Hyg 86: 182. 1440783 10.1016/0035-9203(92)90562-q

[37] Talla I, Kongs A, Verle P, Belot J, Sarr S, et al (1990) Outbreak of intestinal schistosomiasis in the Senegal River Basin. Ann Soc Belg Med Trop 70: 173–180. 2122819

[38] Picquet M, Ernould JC, Vercruysse J, Southgate VR, Mbaye A, et al (1996) Royal Society of Tropical Medicine and Hygiene meeting at Manson House, London, 18 May 1995. The epidemiology of human schistosomiasis in the Senegal river basin. Trans R Soc Trop Med Hyg 90: 340–346. 8882173 10.1016/s0035-9203(96)90501-5

[39] WHO (2006) Preventive chemotherapy in human helminthiasis - Coordinated use of anthelminthic drugs in control interventions: a manual for health professionals and programme managers.

[40] Berger C, Ba N, Gugger M, Bouvy M, Rusconi F, et al (2006) Seasonal dynamics and toxicity of Cylindrospermopsis raciborskii in Lake Guiers (Senegal, West Africa). FEMS Microbiol Ecol 57: 355–366. 16907750 10.1111/j.1574-6941.2006.00141.x

[41] Kitron UD, Higashi GI (1985) Schistosoma haematobium in Upper Egypt: analysis of dispersion patterns. Am J Trop Med Hyg 34: 331–340. 3985275 10.4269/ajtmh.1985.34.331

[42] Richter J, Hatz C, Campagne N, Bergquist R, Jenkins JM (1996) Ultrasound in schistosomiasis: A practical guide to the standardized use of ultrasonography for the assessment of schistosomiasis-related morbidity. TDR/STR/SCH/00.1.

[43] Leutscher PD, Reimert CM, Vennervald BJ, Ravaoalimalala VE, Ramarokoto CE, et al (2000) Morbidity assessment in urinary schistosomiasis infection through ultrasonography and measurement of eosinophil cationic protein (ECP) in urine. Trop Med Int Health 5: 88–93. 10747267 10.1046/j.1365-3156.2000.00522.x

[44] Medhat A, Zarzour A, Nafeh M, Shata T, Sweifie Y, et al (1997) Evaluation of an ultrasonographic score for urinary bladder morbidity in Schistosoma haematobium infection. Am J Trop Med Hyg 57: 16–19. 9242311 10.4269/ajtmh.1997.57.16

[45] Kulldorff M (2010) SaTScan User Guide for version 9.0.

[46] Kulldorff M (1997) A spatial scan statistic. Communications in Statistics - Theory and Methods 26: 1481–1496. 10.1080/03610927708831932 PMC3867306 24363487

[47] Kulldorff M, Huang L, Konty K (2009) A scan statistic for continuous data based on the normal probability model. Int J Health Geogr 8: 58. 19843331 10.1186/1476-072X-8-58 PMC2772848

[48] Jung I, Kulldorff M, Klassen AC (2007) A spatial scan statistic for ordinal data. Stat Med 26: 1594–1607. 16795130 10.1002/sim.2607

[49] Kulldorff M, Mostashari F, Duczmal L, Katherine YW, Kleinman K, et al (2007) Multivariate scan statistics for disease surveillance. Stat Med 26: 1824–1833. 17216592 10.1002/sim.2818

[50] Cook, G C. and Zumla, A I. (2009) Manson's Tropical Diseases. London: Saunders Elsevier.

[51] Farooq M (1966) Importance of determining transmisson sites in planning bilharziasis control. Field observations from the Egypt-49 project area. Am J Epidemiol 83: 603–612. 5934625 10.1093/oxfordjournals.aje.a120611

[52] Polderman AM, Mpamila K, Manshande JP, Gryseels B, Van Schayk O (1985) Historical, geological and ecological aspects of transmission of intestinal schistosomiasis in Maniema, Kivu Province, Zaire. Ann Soc Belg Med Trop 65: 251–261. 3935060

[53] Woolhouse ME, Chandiwana SK (1989) Spatial and temporal heterogeneity in the population dynamics of Bulinus globosus and Biomphalaria pfeifferi and in the epidemiology of their infection with schistosomes. Parasitology 98 (Pt 1) 21–34. 2717216 10.1017/s0031182000059655

[54] Johnson PT, Lund PJ, Hartson RB, Yoshino TP (2009) Community diversity reduces Schistosoma mansoni transmission, host pathology and human infection risk. Proc Biol Sci 276: 1657–1663. 19203926 10.1098/rspb.2008.1718 PMC2660983

[55] Al-Sheikh AH, Dagal MA (2011) The ecological differences between Bulinus beccari, the intermediate host of Schistsoma haematobium and Biomphalaria pfeifferi the intermediate host of S. mansoni in in Jazan Region, Saudi Arabia. J Egypt Soc Parasitol 41: 543–551. 22435148

[56] Ernould JC, Ba K, Sellin B (1999) The impact of the local water-development programme on the abundance of the intermediate hosts of schistosomiasis in three villages of the Senegal River delta. Ann Trop Med Parasitol 93: 135–145. 10474638 10.1080/00034989958618

[57] Amankwa JA, Bloch P, Meyer-Lassen J, Olsen A, Christensen NO (1994) Urinary and intestinal schistosomiasis in the Tono Irrigation Scheme, Kassena/Nankana District, upper east region, Ghana. Trop Med Parasitol 45: 319–323. 7716395

[58] Bethony J, Williams JT, Blangero J, Kloos H, Gazzinelli A, et al (2002) Additive host genetic factors influence fecal egg excretion rates during Schistosoma mansoni infection in a rural area in Brazil. Am J Trop Med Hyg 67: 336–343. 12452486 10.4269/ajtmh.2002.67.336

[59] Grant AV, Araujo MI, Ponte EV, Oliveira RR, Cruz AA, et al (2011) Polymorphisms in IL10 are associated with total Immunoglobulin E levels and Schistosoma mansoni infection intensity in a Brazilian population. Genes Immun 12: 46–50. 20927126 10.1038/gene.2010.50

[60] Grant AV, Araujo MI, Ponte EV, Oliveira RR, Gao P, et al (2012) Functional polymorphisms in IL13 are protective against high Schistosoma mansoni infection intensity in a Brazilian population. PLoS One 7: e35863. 22574126 10.1371/journal.pone.0035863 PMC3345031

[61] Lima e Costa MF, Magalhaes MH, Rocha RS, Antunes CM, Katz N (1987) Water-contact patterns and socioeconomic variables in the epidemiology of schistosomiasis mansoni in an endemic area in Brazil. Bull World Health Organ 65: 57–66. 3107847 PMC2490852

[62] Farooq M, Nielsen J, Samaan SA, Mallah MB, Allam AA (1966) The epidemiology of Schistosoma haematobium and S. mansoni infections in the Egypt-49 project area. 2. Prevalence of bilharziasis in relation to personal attributes and habits. Bull World Health Organ 35: 293–318. 5297627 PMC2476086

[63] Kloos H, Gazzinelli A, Van Zuyle P (1998) Microgeographical patterns of schistosomiasis and water contact behavior; examples from Africa and Brazil. Mem Inst Oswaldo Cruz 93 Suppl 1: 37–50. 9921322 10.1590/s0074-02761998000700006

[64] Brouwer KC, Ndhlovu PD, Wagatsuma Y, Munatsi A, Shiff CJ (2003) Epidemiological assessment of Schistosoma haematobium-induced kidney and bladder pathology in rural Zimbabwe. Acta Trop 85: 339–347. 12659971 10.1016/s0001-706x(02)00262-0

[65] Brouwer KC, Munatsi A, Ndhlovu PD, Wagatsuma Y, Shiff CJ (2004) Urinary schistosomiasis in Zimbabwean school children: predictors of morbidity. Afr Health Sci 4: 115–118. 15477190 PMC2141624

[66] El Khoby T, Galal N, Fenwick A, Barakat R, El Hawey A, et al (2000) The epidemiology of schistosomiasis in Egypt: summary findings in nine governorates. Am J Trop Med Hyg 62: 88–99. 10813505 10.4269/ajtmh.2000.62.88

[67] Garba A, Pion S, Cournil A, Milet J, Schneider D, et al (2010) Risk factors for Schistosoma haematobium infection and morbidity in two villages with different transmission patterns in Niger. Acta Trop 115: 84–89. 20171156 10.1016/j.actatropica.2010.02.007

[68] Heurtier Y, Lamothe F, Develoux M, Docquier J, Mouchet F, et al (1986) Urinary tract lesions due to Schistosoma haematobium infection assessed by ultrasonography in a community based study in Niger. Am J Trop Med Hyg 35: 1163–1172. 3098123 10.4269/ajtmh.1986.35.1163

[69] Serieye J, Boisier P, Ravaoalimalala VE, Ramarokoto CE, Leutscher P, et al (1996) Schistosoma haematobium infection in western Madagascar: morbidity determined by ultrasonography. Trans R Soc Trop Med Hyg 90: 398–401. 8882187 10.1016/s0035-9203(96)90521-0

[70] Traore M, Traore HA, Kardorff R, Diarra A, Landoure A, et al (1998) The public health significance of urinary schistosomiasis as a cause of morbidity in two districts in Mali. Am J Trop Med Hyg 59: 407–413. 9749635 10.4269/ajtmh.1998.59.407

[71] King CH, Lombardi G, Lombardi C, Greenblatt R, Hodder S, et al (1988) Chemotherapy-based control of schistosomiasis haematobia. I. Metrifonate versus praziquantel in control of intensity and prevalence of infection. Am J Trop Med Hyg 39: 295–305. 3140683 10.4269/ajtmh.1988.39.295

[72] Southgate VR, De Clercq D, Sene M, Rollinson D, Ly A, et al (2000) Observations on the compatibility between Bulinus spp. and Schistosoma haematobium in the Senegal River basin. Ann Trop Med Parasitol 94: 157–164. 10827870 10.1080/00034980057491

[73] King CH, Muchiri EM, Ouma JH (1992) Age-targeted chemotherapy for control of urinary schistosomiasis in endemic populations. Mem Inst Oswaldo Cruz 87 Suppl 4: 203–210. 1343896 10.1590/s0074-02761992000800031

[74] Bethony JM, Quinnell RJ (2008) Genetic epidemiology of human schistosomiasis in Brazil. Acta Trop 108: 166–174. 18207118 10.1016/j.actatropica.2007.11.008

[75] Gong YY, Wilson S, Mwatha JK, Routledge MN, Castelino JM, et al (2012) Aflatoxin exposure may contribute to chronic hepatomegaly in Kenyan school children. Environ Health Perspect 120: 893–896. 22370114 10.1289/ehp.1104357 PMC3385435

[76] Booth M, Vennervald BJ, Kenty L, Butterworth AE, Kariuki HC, et al (2004) Micro-geographical variation in exposure to Schistosoma mansoni and malaria, and exacerbation of splenomegaly in Kenyan school-aged children. BMC Infect Dis 4: 13. 15147584 10.1186/1471-2334-4-13 PMC421731

[77] WHO (2013) Schistosomiasis: progress report 2001–2011, strategic plan 2012–2020.

[78] WHO (2011) Helminth control in school-age children: A guide for managers of control programmes. WHO Library Cataloguing-in-Publication Data.

[79] Sturrock RF (1989) The control of schistosomiasis: epidemiological aspects of reinfection. Mem Inst Oswaldo Cruz 84 Suppl 1: 134–148. 2700590 10.1590/s0074-02761989000500014

[80] Peng WX, Tao B, Clements A, Jiang QL, Zhang ZJ, et al (2010) Identifying high-risk areas of schistosomiasis and associated risk factors in the Poyang Lake region, China. Parasitology 137: 1099–1107. 20128946 10.1017/S003118200999206X

